# mTOR inhibitor efficacy is determined by the eIF4E/4E-BP ratio

**DOI:** 10.18632/oncotarget.799

**Published:** 2012-12-31

**Authors:** Tommy Alain, Nahum Sonenberg, Ivan Topisirovic

**Affiliations:** Department of Biochemistry and Goodman Cancer Centre, McGill University Montreal, Quebec, Canada; Department of Biochemistry and Goodman Cancer Centre, McGill University Montreal, Quebec, Canada; Lady Davis Institute for Medical Research, SMBD-Jewish General Hospital and Department of Oncology, McGill University Montreal, Quebec, Canada

The mammalian/mechanistic target of rapamycin (mTOR) is a multifunctional serine/threonine kinase that is hyperactivated in cancer [1]. mTOR forms two distinct complexes, mTORC1 and 2. mTORC1 stimulates translation and perturbs energy metabolism to drive cell proliferation and growth, whereas mTORC2 regulates cytoskeletal organization and cell survival by stimulating AGC kinases (e.g. AKT). Therefore, suppressing mTOR's activity is widely considered as a very attractive anti-cancer therapy. Accordingly, mTOR inhibitors are increasingly used as anti-neoplastic agents in the clinic.

Rapamycin is a naturally occurring allosteric inhibitor of mTORC1 and its analogs (rapalogs) are used as anti-cancer agents for the treatment of refractory renal cell carcinomas, mantle cell lymphomas, and pancreatic neuroendocrine tumors. However, success of rapalogs as anti-cancer monotherapies is limited [2]. This has been attributed to the activation of AKT signaling resulting from the loss of a negative-feedback mechanism, as well as rapamycin-resistant mTORC1 outputs, such as the phosphorylation of the eukaryotic initiation factor 4E-binding proteins (4E-BPs).

To overcome these deficiencies a new generation of ATP-competitive mTOR inhibitors [also referred to as dual mTORC^1^/2 inhibitors or active-site mTOR inhibitors (asTORi)] was developed [2]. asTORi suppress AKT signaling by inhibiting mTORC2, and abrogate rapamycin-resistant mTORC1 outputs including the phosphorylation of 4E-BPs. Accordingly, asTORi exhibit stronger anti-proliferative and anti-tumorigenic effects as compared to rapalogs, and are currently in multiple clinical trials aiming to target aberrant mTOR signaling in cancer [2]. However, a major obstacle to applying asTORi to the clinic is a lack of predictive biomarkers that would facilitate the stratification of the patients that are most likely to respond to these drugs. Discovery of predictive biomarkers indicative of the efficacy of mTOR inhibitors is hampered by the complexity of the mTOR pathway, given that mTOR controls a variety of cellular processes via a multitude of substrates.

**Figure 1:**
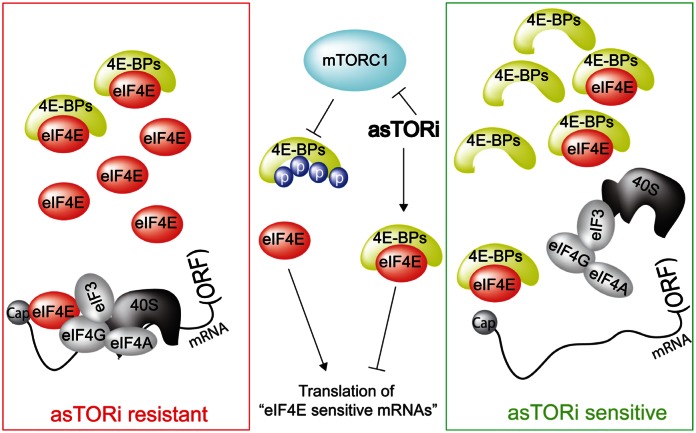
Sensitivity of tumor to asTORi as a function of eIF4E/4E-BP ratio mTORC1 phosphorylates and inactivates 4E-BPs, thereby stimulating translation of “eIF4E-sensitive” mRNAs and driving cell proliferation and growth. asTORi abrogate the phosphorylation of 4E-BPs by mTORC1 leading to reduction in translation of “eIF4E-sensitive” mRNAs. However, in cancer cells with elevated eIF4E/4E-BP ratio (red rectangle), inhibition of translation of “eIF4E-sensitive” mRNAs by asTORi is incomplete and insufficient to suppress neoplastic growth. In turn, in malignant cells exhibiting low eIF4E/4E-BP ratio (green rectangle), asTORi abolish “eIF4E-sensitive” mRNA translation and suppress neoplastic growth. ORF: open reading frame.

4E-BPs are a family of small translational repressors, which sequester the 5' mRNA cap-binding protein eukaryotic translation initiation factor (eIF)-4E (eIF4E), thereby impeding the assembly of the eIF4F complex. The eIF4F complex, which consists of eIF4E, scaffolding protein eIF4G and the DEAD-box helicase eIF4A, recruits the mRNA to the ribosome to initiate translation. mTORC1 phosphorylates and inactivates 4E-BPs, thereby facilitating the assembly of the eIF4F complex and translation initiation (See Figure 1). eIF4E acts as a general translation initiation factor, but a subset of mRNAs referred to as “eIF4E-sensitive” are particularly sensitive to changes in eIF4E activity. These mRNAs encode tumor-promoting factors, such as cyclin D3, ornithine decarboxylase and myc. The inability of rapalogs to completely suppress 4E-BP phosphorylation and translation of “eIF4E-sensitive” mRNAs is thought to be the reason for their relatively limited anti-tumorigenic efficacy in the clinic [2, 3].

Overexpression of eIF4E and variations in 4E-BP levels and phosphorylation are frequently observed in tumors, thereby suggesting that eIF4E/4E-BP stoichiometry may significantly differ among patients, or even within a single tumor [4]. We demonstrated that the eIF4E/4E-BP ratio determines the ability of asTORi to suppress neoplastic growth [5]. Resistance of malignant cells with high eIF4E/4E-BP ratio to asTORi can be explained by their deficiency to inhibit eIF4F complex assembly and translation of “eIF4E-sensitive” mRNAs (Figure 1). We also showed that cancer cells acquire resistance to asTORi by increasing eIF4E availability via downregulation of 4E-BP1 and 2. These data corroborate earlier findings showing that the amplification of the eIF4E gene underlies resistance to the dual PI3K/mTOR inhibitor BEZ235 [6], and that eIF4E translation activity can predict sensitivity to rapalogs [3]. Therefore, eIF4E/4E-BP ratio, rather than individual levels or phosphorylation status of these proteins, is more likely to serve as a prognostic biomarker to select the patients for clinical trials using asTORi and to tailor personalized mTOR targeted therapies.

Recently, several mechanisms potentiating resistance to mTOR inhibitors emerged, including the activation of alternative signaling pathways such as the MAPK pathway [7]. Therefore, combined targeting of the mTOR and MAPK pathways has been suggested as a promising approach to overcome resistance to mTOR inhibitors in the clinic. In addition to this strategy, our findings suggest that combining therapeutic approaches that suppress eIF4E expression or activity (e.g. ISIS-EIF4ERx, 4EGI-1, or 4E1RCat [8]) with mTOR targeted therapies should be beneficial in patients bearing tumors with elevated eIF4E/4E-BP ratio.
